# Identifying stage-specific protein subnetworks for colorectal cancer

**DOI:** 10.1186/1753-6561-6-S7-S1

**Published:** 2012-11-13

**Authors:** Sinan Erten, Salim A Chowdhury, Xiaowei Guan, Rod K Nibbe, Jill S Barnholtz-Sloan, Mark R Chance, Mehmet Koyutürk

**Affiliations:** 1 Department of Electrical Engineering & Computer Science, Case Western Reserve University, Cleveland, OH, USA; 2 School of Computer Science, Carnegie Mellon University, Pittsburgh, PA, USA; 3 Case Center for Proteomics & Bioinformatics, Case Western Reserve University, Cleveland, OH, USA; 4 Department of Epidemiology and Biostatistics, Case Western Reserve University, Cleveland, OH, USA; 5 Case Comprehensive Cancer Center, Case Western Reserve University, Cleveland, OH, USA; 6 Department of Genetics, Case Western Reserve University, Cleveland, OH, USA

## Abstract

**Background:**

In recent years, many algorithms have been developed for network-based analysis of differential gene expression in complex diseases. These algorithms use protein-protein interaction (PPI) networks as an integrative framework and identify subnetworks that are coordinately dysregulated in the phenotype of interest.

**Motivation:**

While such dysregulated subnetworks have demonstrated significant improvement over individual gene markers for classifying phenotype, the current state-of-the-art in dysregulated subnetwork discovery is almost exclusively limited to binary phenotype classes. However, many clinical applications require identification of molecular markers for multiple classes.

**Approach:**

We consider the problem of discovering groups of genes whose expression signatures can discriminate multiple phenotype classes. We consider two alternate formulations of this problem (i) an all-vs-all approach that aims to discover subnetworks distinguishing all classes, (ii) a one-vs-all approach that aims to discover subnetworks distinguishing each class from the rest of the classes. For the one-vs-all formulation, we develop a set-cover based algorithm, which aims to identify groups of genes such that at least one gene in the group exhibits differential expression in the target class.

**Results:**

We test the proposed algorithms in the context of predicting stages of colorectal cancer. Our results show that the set-cover based algorithm identifying "stage-specific" subnetworks outperforms the all-vs-all approaches in classification. We also investigate the merits of utilizing PPI networks in the search for multiple markers, and show that, with correct parameter settings, network-guided search improves performance. Furthermore, we show that assessing statistical significance when selecting features greatly improves classification performance.

## Introduction

Genome-wide monitoring of mRNA expression, monitored using DNA microarrays and more recently deep sequencing, has proved quite useful in understanding the mechanistic bases of complex human diseases. Systematic analysis of differential gene expression in different phenotypic classes leads to identification of novel biomarkers, which serve as features for phenotype classification, as well as targets for therapeutic intervention. In previous studies, differential analysis of gene expression led to identification of biomarkers for a range of complex diseases, including Parkinson's disease [1], neuroblastoma [2], lung cancer [3] and breast cancer [4].

Traditional analyses generally take a univariate approach to study gene expression and identify genes with significant individual differential expression in the phenotype of interest. However, such univariate approaches are often limited in explaining the underlying mechanisms of complex diseases, which arise from the interplay among multiple genetic and environmental factors. For example, genes that cooperate or complement each other in pathogenesis may not necessarily be differentially expressed individually, but exhibit coordinated dysregulation when considered together.

In order to address the shortcomings of the univariate approaches, Chuang *et al*. develop an algorithm that integrates gene expression data with protein-protein interaction (PPI) networks to identify reproducible breast cancer metastasis markers composed of multiple interacting proteins ("dysregulated subnetworks") [5]. They show that these subnetwork markers better predict breast cancer metastasis as compared to individually dysregulated genes. Motivated by the demonstrated promise of this approach, several other algorithms are developed for network-based analysis of differential gene expression. In particular, Chowdhury *et al*. develop a set-cover based heuristic for identification of genes that complement each other in discriminating phenotype and control samples [6]. Phuong *et al*. further improve on these algorithms by introducing a biclustering algorithm that also accounts for the noise in PPI networks by incorporating reliability scores for PPIs [7]. More recently, recognizing the shortcomings of greedy algorithms in identifying dysregulated subnetworks, Phuong *et al*. introduce a color-coding based randomized algorithm to identify subnetworks that are highly discriminative of phenotype and control [8]. These methods are also extended to the identification of subnetwork expression signatures that can shed light into the regulatory logic of the relationship between the dysregulation of multiple genes and the disease phenoype. In particular, Chowdury *et al*. identify subnetworks whose combinatorial expression states are indicative of phenotype by using a branch-and-bound algorithm [9], Dutkowski *et al*. grow network-guided forests by training decision trees using interacting proteins [10].

All of the existing dysregulated subnetwork discovery algorithms are designed and validated for binary phenotype classes (*e.g*. cancerous *vs*. non-cancerous, metastatic *vs*. non-metastatic, drug responders *vs*. non-responders) and prove to be promising in terms of accurate classification of samples. However, many progressive diseases such as glioblastoma, breast cancer and colorectal cancer require identification of molecular markers for multiple classes (such as the four stages in colorectal cancer according to Dukes' classification) for effective prognosis and treatment. This implies the necessity of a framework that can also work on datasets with more than two phenotype classes for network-based discovery of disease markers. Although most of the existing algorithms can be applied to multiple phenotype classes in principle, no tool is readily available for this purpose. Furthermore, subnetwork discovery on multi-class datasets requires additional design choices and poses novel algorithmic challenges. These choices include designing criteria to evaluate the dysregulation of a subnetwork; *i.e*., are we interested in identifying subnetworks that can distinguish all classes from each other at once, or are we interested in identifying subnetworks that serve as indicators for specific classes. The algorithmic challenges, on the other hand, include unproportionately distributed samples across multiple classes. For these reasons, novel algorithms are needed that are robust and can work with datasets that are composed of different number of classes and sample distributions.

## Contributions of this study

In this article, we introduce novel algorithms for network-based analysis of differential gene expression on applications that involve multiple phenotype classes. As an important application, we focus particularly on identifying subnetworks that can discriminate different stages of human colorectal cancer (CRC) according to Dukes' classification. We first propose two formulations to generalize information-theoretic measures of subnetwork dysregulation to multiple phenotype classes. These formulations differ in terms of how the target subnetworks discriminate phenotype classes from each other; namely we establish information-theoretic criteria for *all-vs-all *and *one-vs-all *discriminative subnetworks. Then, we extend the set-cover based algorithm by Chowdury *et al*. , NETCOVER, to identify *one-vs-all *discriminative subnetworks [6]. We also introduce a framework for assessing the statistical significance of the sub-networks identified by the set-cover based algorithm. Using public CRC datasets composed of samples labeled with Dukes' four stages, we investigate the performance of the resulting algorithm, COBALT, in identifying subnetworks that are useful in predicting the stages of colon cancer samples. In particular we perform systematic computational experiments to investigate the following:

• We compare the performance of *all-vs-all *and *one-vs-all *subnetworks in predicting phenotype and show that *one-vs-all *discriminative subnetworks are generally more reliable as features for classification.

• We investigate the effect of using the PPI network to confine the space for searching groups of genes that are coordinately dysregulated subnetworks. We show that, while expansion of the search space through consideration of indirect interactions improve the classification performance of identified subnetworks, this improvement saturates after a point, demonstrating that PPI networks indeed provide a shortcut to the identification of dysregulated groups of genes. We also show that our efficient set cover based algorithm renders network-free search feasible.

• We investigate the effect of using statistically significant subnetworks (as opposed to high-scoring subnetworks) as features for classification and show that assessment of statistical significance facilitates identification of more useful subnetwork features for classification.

In the next section, we start our discussion by proposing two alternate information-theoretic formulations of sub-network dysregulation. We also introduce our set-cover based algorithm, COBALT, for the identification of *one-vs-all *discriminative subnetworks and propose methods for assessing the statistical significance of the identified subnetworks. Subsequently, in Results Section, we provide comprehensive experimental results on the classification performance of the subnetworks discovered by COBALT in predicting the stage of CRC on two gene expression datasets obtained from the Gene Expression Omnibus. We conclude the paper in Conclusion Section.

## Methods

In this section, we start by introducing the mathematical background of the information-theoretic formulation of coordinate dysregulation for a set of genes. Subsequently, we propose two alternate approaches for generalizing this notion to multiple phenotype classes. We then introduce COBALT, our set-cover based algorithm that is specifically designed to identify stage-specific discriminative subnetworks. Finally, we introduce a framework for assessing the statistical significance of the identified subnetworks, and describe how these subnetworks can be utilized for classification of samples.

### Dysregulation of subnetworks

For a given set  of genes and  of samples, let *E_i _*∈ *R*^|*u*| ^represent the properly normalized gene expression vector for gene $g_{i}\in V$, where *E_i_*(*j*) denotes the relative expression of *g_i _*in sample $s_{j}\in U$. Assume that we have a set , composed of different classes for the phenotype of interest (such as the four stages in colorectal cancer according to Dukes classification) and the phenotype vector *C *annotates each sample with one of the labels in  , i.e., *C*(*j*) = *t *where *t *∈  .We also define the set of all samples for a specific phenotype class *t *as $U^{\left(t\right)}=\left\{s_{j}\in U:C\left(j\right)=t\right\}$.

Let G(V,ε) denote a PPI network where the product of each gene *g_i _*∈  is represented by a node and each edge *g_i_g_j _*represents an interaction between the products of *g_i _*and *g_j_*. Given a PPI network and a gene expression dataset over multiple phenotype classes, we are interested in finding sets of genes that can together discriminate the phenotype classes with their gene expression signatures. In order to establish the functional relevance of these gene sets and search for these sets more efficiently, we confine the search space to PPI subnetworks, that is groups of proteins that are functionally interrelated through PPIs. Formally, a set S⊆V of proteins is considered a subnetwork of interest if for all proteins *g_i _*∈ , there is at least one other protein *g_j _*∈ such that *g_i _*and *g_j _*are connected through at most ℓ hops in the PPI network. Here, ℓ is a parameter that adjusts the trade-off between functional relevance and computational efficiency; a larger ℓ allows searching for functionally less related proteins at the cost of increasing the search space.

For a given subnetwork S⊆V, Chuang *et al*. define the *subnetwork activity *of as $E_{S}=\sum_{g_{i}\in S}E_{i}/\sqrt{\left|S\right|}$, that is the aggregate expression profile of the genes in [5]. Using subnetwork activity, they define an information-theoretic measure to quantify the dysregulation of a subnetwork. This "additive" definition of dysregulation limits the framework to the identification of subnetworks with all genes in the subnetwork dysregulated in the same direction (*i.e*., all up- or down-regulated in the phenotype of interest), and alternate approaches that compute combinatorial expression signatures are shown to be more powerful [9,10]. However, this additive formulation serves as a useful starting point to generalize subnetwork dysregulation to phenotypes that involve multiple classes. For this reason, we focus on additive subnetwork activity in this paper.

#### All-vs-all discriminative power of a subnetwork

It is straightforward to generalize the information-theoretic measure for the dysregulation of a subnetwork [5] to multiple phenotype classes. Namely, the mutual information between the subnetwork activity of *S *and the multi-class phenotype vector, *i.e*., Δ _all-vs-all _(*S*) = *I *(*E_S_*, *C*) = *H*(*C*) − *H*(*C*|*E_S _*), provides a measure of the the reduction in the uncertainty about *C *given *E_S_*. Here, $H\left(X\right)=-\sum_{x\in \chi }p\left(x\right)\;\text{log}\left(p\left(x\right)\right)$ denotes the Shannon entropy of discrete random variable *X *that can take over values from the set  . In our case, the support set for the random variable *C *is , whereas the support set for the random variable *E_S _*is obtained by appropriately quantizing the expression levels.

#### One-vs-all discriminative power of a subnetwork

Here, we propose an alternate measure to quantify the power of a subnetwork in discriminating multiple phenotype classes from each other. This measure targets discriminating a particular phenotype class from all other classes. Namely, we define class-specific phenotype vector *C*^(*t*) ^for class *t *∈  as

(1) $$c_{j}^{\left(t\right)}=\{\begin{array}{ll} 1 & \text{if}\;C_{j}=t\\ 0 & \text{otherwise}\text{.} \end{array}$$

**Figure 1:**
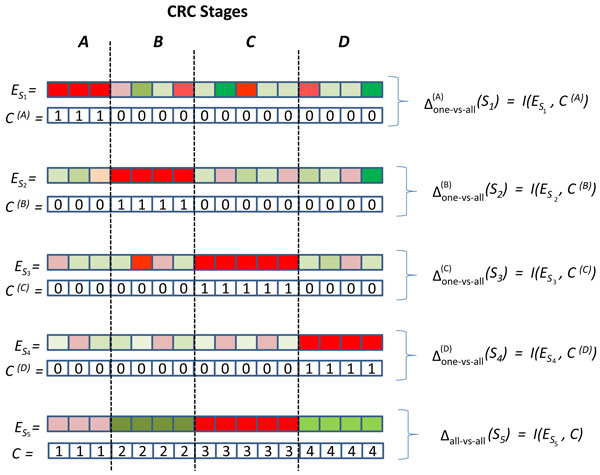
**Illustration of the difference between all-vs-all and one-vs-all discriminative subnetworks**. Illustration of the difference between all-vs-all and one-vs-all discriminative subnetworks. The aggregate expression profiles of five hypothetical subnetworks are shown. Red and green respectively represent positive and negative expression, with intensity representing magnitude. The subnetworks $S_{1},S_{2},S_{3}$ and $S_{4}$ are one-vs-all discriminative, respectively indicating classes (CRC stages) A, B, C and D, since the expression profile of each subnetwork in samples that belong to the respective class can discriminate these samples from other classes. On the other hand, $S_{5}$ is an all-vs-all discriminative subnetwork, since it discriminates all classes from each other.

Then, the mutual information between subnetwork activity and the class-specific phenotype vector *C*^(*t*)^, *i.e*., $\Delta_{\text{one - vs - all}}^{\left(t\right)}\;\left(S\right)=I\left(E_{S},C^{\left(t\right)}\right)$, provides a measure of the reduction in the uncertainty about class *t *given *E_S_*. This formulation offers a number of benefits as compared to the all-vs-all formulation of discriminative power: (1) The one-vs-all formulation may lead to identification of more interpretable markers, since, for example, it can provide stage-specific molecular signatures for colorectal cancer. (2) The one-vs-all formulation can extract class-specific molecular signatures that may be missed by the all-vs-all formulation because they do not discriminate other classes well. (3) The phenotype random variable takes a smaller number of values, thus offering more statistical power for the same number of samples. The concepts of all-vs-all and one-vs-all disciminative power of subnetworks and the computation of mutual information using these different formulations are illustrated in Figure 1.

### Identifying one-vs-all discriminative subnetworks

The problems of identifying subnetworks with maximum $\Delta_{\text{all - vs - all}}\left(S\right)$ or $\Delta_{\text{one - vs - all}}\left(S\right)$ are intractable [5]. However, it is straightforward to generalize the greedy algorithm by Chuang *et al*. to solve both problems efficiently [5]. This greedy algorithm initializes a subnetwork with a single protein. It then grows the subnetwork by adding the protein in the neighborhood of the subnetwork (*i.e*., reachable from the subnetwork with ℓ hops) that improves the objective function ($\Delta_{\text{all - vs - all}}\left(S\right)$ or $\Delta_{\text{one - vs - all}}\left(S\right))$ the most. The algorithm stops either when there is no more protein in the neighborhood to add, or the best improvement provided by a protein in the neighborhood is below a user-defined threshold.

While the explained greedy algorithm is quite effective in efficiently discovering high-scoring subnetworks, it has several drawbacks [6]. First, this algorithm is biased toward identifying subnetworks with very few proteins that exhibit high dysregulation individually. This is because the algorithm lacks global awareness, *i.e*., it will stop expanding the subnetwork when the best candidate protein to add to the subnetwork has only marginal individual contribution, but may actually contribute a greater deal when additonal proteins are added. Second, this approach requires computation of mutual information for each and every candidate protein in the neighbourhood to be added to the growing subnetwork, which may prove to be costly when the algorithm needs to be run multiple times to assess statistical significance of identified subnetworks. Motivated by these observations, Chowdhury *et al*. develop a set-cover based algorithm, NETCOVER, which is more effective in discovering proteins that complement each other in discriminating phenotype and control samples [6]. However, NETCOVER is designed for binary phenotype classes and it assumes that the samples are paired. Here, we argue that the algorithmic insights introduced by NETCOVER suit particularly well to the identification of one-vs-all discriminative subnetworks. Based on this observation, we develop COBALT, which generalizes NETCOVER to handle unpaired samples and multiple phenotype classes to identify one-vs-all discriminative subnetworks.

### COBALT:Cover-based algorithm for identifying one-vs-all discriminative subnetworks

Recall that a one-vs-all discriminative subnetwork is defined as one with differential subnetwork activity in a specific phenotype class, as compared to all other classes. Since subnetwork activity is defined regularly, the genes in such a subnetwork have to be either all up-regulated or all down-regulated in the phenotype class of interest. Motivated by this observation, COBALT aims to identify subnetworks such that for each sample that belongs to the phenotype class of interest, there exists at least one gene in the subnetwork that is up-regulated (or down-regulated) in that sample. Such subnetworks are said to "cover" the entire patient population that represents the phenotype class of interest.

In order to identify the genes that are up-regulated or down-regulated in each sample, we use the expression of that gene in all samples as the background distribution. Subsequently, we identify samples in which the genes' expression deviates significantly from this background distribution. Namely, for a gene *g_i_*, consider the distribution of the expression values *E_i _*across all samples. We compute a quantized expression value for *g_i _*in sample *s_j _*as follows:

(2) $${\hat{\text{E}}}_{i}\left(j\right)=\left\{\begin{array}{cc} +1 & \text{if}\;E_{i}\left(j\right)>\mu_{i}+\alpha *\sigma_{i}\\ -1 & \text{if}\;E_{i}\left(j\right)<\mu_{i}-\alpha *\sigma_{i}\\ 0 & \text{otherwise} \end{array}\right)$$

**Figure 2:**
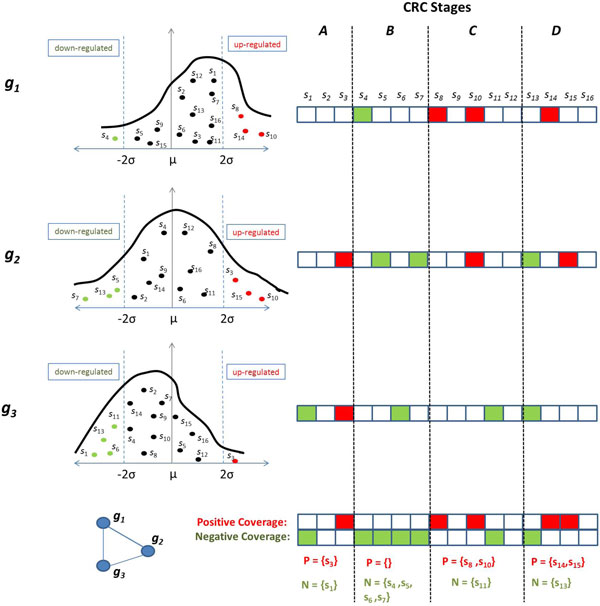
**Illustration of the set-cover based algorithm for the identification of one-vs-all discriminative subnetworks**. Here, the coverage provided by a hypothetical three-gene subnetwork is shown. In the left panel, the distribution of the expression levels of each gene across all samples is shown. We first compute the mean (*μ_i_*) and the standard deviation (*σ_i_*) of this distribution for each gene *g_i_*. Subsequently, we identify samples that are positively or negatively covered by each gene. A gene *g_i _*with expression greater than *μ_i _*+ α * *σ_i _*in a sample is said to positively cover that sample, while a gene *g_i _*with expression less than *μ_i _*- α * *σ_i _*in a sample is said to negatively cover that sample (we set *α *= 2 in our experiments). The negative and positive cover sets for each gene and the subnetwork composed of *g*_1_, *g*_2 _and *g*_3 _are shown on the right. In this example, this subnetwork negatively covers (all samples in) Stage B.

Here, *μ_i _*and *σ_i _*respectively represent the mean and standard deviation of expression value of *g_i _*across all samples, *i.e*., $\mu_{i}=\sum_{S_{j}\in U}E_{i}\left(j\right)/|U|$ and $\sigma_{i}=\sqrt{\sum_{S_{j}\in U}{\left(E_{i}\left(j\right)-\mu_{i}\right)}^{2}}$. We define *α *as a user-defined threshold parameter for a gene's dysregulation in a sample to be considered significant. We say that a gene *g_i _positively covers *a sample *s_j _*if ${\hat{\text{E}}}_{i}\left(j\right)=+1$, and *negatively covers s_j _*if ${\hat{\text{E}}}_{i}\left(j\right)=-1$, Following this definition, for a given gene *g_i _*and phenotype class *t *∈ , we define the positive cover set $P_{i}^{\left(t\right)}$ as the set of all samples with phenotype *t *that are positively covered by *g_i_*, *i.e*., $P_{i}^{\left(t\right)}=\left\{s_{j}\in U:C\left(j\right)=t\;\;\text{and}\;{\hat{\text{E}}}_{i}\left(j\right)=+1\right\}$. Similarly, the negative cover set $N_{i}^{\left(t\right)}$ contains all samples in class *t *that are negatively covered by gene *g_i_*. We illustrate these concepts in Figure 2. It can be shown that the mutual information of *C*^(*t*) ^and *Ê_i _*is a monotonically non-decreasing function of the cardinalities of $P_{i}^{\left(t\right)}$ and $N_{i}^{\left(t\right)}$[6], *i.e*., one can maximize *I*(*Ê_i_*,*C*^(*t*)^) by maximizing $\left||P_{i}^{\left(t\right)}|-|N_{i}^{\left(t\right)}|\right|$.

Using the negative and positive cover sets for each gene-class pair, COBALT identifies one-vs-all discriminative subnetworks for each phenotype class by using a greedy heuristic that is shown to be effective for the set-cover problem [11]. Namely, for each gene *g_i_*, we first identify the target phenotype class for that gene as the phenotype class with largest percentage of samples positively (or negatively) covered by *g_i_*. Subsequently, we grow a subnetwork by systematically adding a gene in the neighborhood based on the coverage on the rest of the uncovered samples for that class. Without loss of generality, the algorithm identifies a minimal positive covering subnetwork seeded at gene *g_i _*as follows:

1. Initialize subnetwork: *S_i _*← {*g_i_*}

2. Define the target phenotype class *t *for this subnetwork as the class that has the maximum fraction of samples positively covered by *g_i_*: *t *← argmax*_t_*′ _∈_$\left\{\left|P_{i}^{\left(t^{\prime }\right)}|/|U_{i}^{\left(t^{\prime }\right)}\right|\right\}$

3. Initialize the set of uncovered samples for class *t*: $M^{\left(t\right)}\leftarrow U^{\left(t\right)}\backslash P_{i}^{\left(t\right)}$

4. Initialize the set of network neighbors: $Q\leftarrow \left\{g_{j}\in V:\delta \left(g_{i},g_{j}\right)\leq \ell \right\}$

5. For all genes *g_j _*∈ , compute ${\left(P_{j}^{\left(t\right)}\right)}^{\prime }\leftarrow P_{j}^{\left(t\right)}\cap M^{\left(t\right)}\text{and }{\left(N_{j}^{\left(t\right)}\right)}^{\prime }\leftarrow N_{j}^{\left(t\right)}\cap M^{\left(t\right)}$

6. Find all the genes (can be multiple) in  with maximum $\left|{\left(P_{j}^{\left(t\right)}\right)}^{\prime }\left|-\right|{\left(N_{j}^{\left(t\right)}\right)}^{\prime }\right|$ and let *g_k _*be the gene among these genes with minimum $\sum_{{}_{{}_{t^{\prime }\in T}\backslash \left\{t\right\}}}\left|P_{k}^{{\left(t\right)}^{\prime }}\right|$(*i.e*., *g_k _*has minimum positive background coverage).

7. Expand the subnetwork with *g_k_*: *S_i _*← *S_i _*∪ {*g_k_*}

8. Update the set of uncovered positive samples for class *t*: *ℳ *^(*t*) ^← *ℳ *^(*t*) ^\ *_k_*^(*t*)^

9. Update set of neighbouring genes:  ←  ∪ {*g_j _*∈  : *δ*(*g_k_*, *g_j_*) ≤ *ℓ*} \ {*g_k_*}

10. If  = ∅ or *M*^(*t*) ^= ∅, return *S_i_*; otherwise, go to step (5).

This algorithm is also used for identifying the minimal negative covering subnetwork seeded at *g_i _*by simply replacing  with  above.

### Assessing statistical significance of subnetworks

In order to asses the significance of the identified subnetworks, we perform two distinct significance tests. Each significance test is performed by generating an empirical background distribution that carefully accounts for multiple hypothesis testing. The first background distribution is obtained by randomly permuting the class labels. The second background distribution, on the other hand, is obtained by permuting the gene expression profiles (the rows of the gene expression matrix, thereby randomly reestablishing the relationship between the expression profiles and the nodes in the PPI network ). After generating a large number of these randomized datasets, we use COBALT to identify class-specific subnetworks for the randomized datasets as well. We then use these subnetworks as the background distribution to test the statistical significance of the discriminative power of the stage-specific subnetworks identified on the actual dataset. This approach implicitly handles multiple hypothesis testing, since the background distribution is constructed using the most discriminative subnetworks that could be identified on each randomized dataset.

Note also that the cover provided by a subnetwork for a target phenotype class depends on the size of the sub-network (*i.e*., the number of proteins in the subnetwork). In other words, if we construct subnetworks at random, we would expect larger subnetworks to have a higher coverage. Furthermore, in our experiments, we also observe that larger subnetworks tend to have higher discriminative power (Δ ). Motivated by these insights, we assess the statistical significance of a subnetwork as a function of its size. For this purpose, we stratify the subnetworks that compose each background distribution according to subnetwork size and compute the *p*-value of a subnetwork *S *by comparing Δ(*S*) to the discriminative power of the background subnetworks that have similar size to the subnetwork of interest. More precisely, the *p*-value of *S *is defined as the fraction of subnetworks with discriminative power greater than that of *S *among all subnetworks in the background set with size equal to that of *S*. A minimal covering subnetwork *S *discovered by COBALT is considered to be statistically significant if its *p*-value is less than the significance threshold for both background populations.

### Using identified subnetworks for classification

One application of identifying subnetworks that can discriminate multiple phenotype classes is to predict the phenotype class of a test sample using the expression profiles of these subnetworks. This application also provides a useful means for assessing the biological relevance, reproducibility, and utility of the identified subnetworks. In order to use stage-specific subnetworks in colorectal cancer to predict the stage of a patient, following steps are performed:

1. We first identify both the positive and negative covering subnetworks for each gene *g_i _*∈ .

2. In order to investigate the effect of statistical significance on the classification utility of subnetworks, we use two alternate strategies to extract a list of features from the sets of covering subnetworks found in (1):

(a) The first approach assumes that high-scoring subnetworks are more useful for classification, as compared to significantly discriminative subnetworks. For each phenotype class *t *∈ , we sort the subnetworks based on their all-vs-all $\left(\Delta_{\text{all}-\text{vs}-\text{all}}\left(S\right)\right)$ or one-vs-all $\left(\Delta_{\text{all}-\text{vs}-\text{all}}\left(S\right)\right)$ discriminative power. We then choose the top *k *positive and negative covering subnetworks for each phenotype class, giving us a total of 2*k*|| features to be used in classification. Here, *k *is a user defined parameter to set the number of stage-specific subnetworks that are used for each class.

(b) The second approach assumes that assessment of statistical significance will facilitate selection of biologically more meaningful subnetworks, also providing more power in classification as compared to high-scoring subnetworks. For this purpose, using the two proposed statistical significance tests discussed in the previous section, we identify subnetworks that are signficant according to both statistical tests and use all of these significantly discriminative subnetworks as features for classification.

3. Once we obtain a final list of features either using (a) or (b) at step (2), we compute the aggregate expression profiles (*E_S _*) for each of these selected subnetworks and use these to construct feature vectors for each sample (where each feature represents the aggreate expression of one subnetwork).

4. Finally, we use these feature vectors to train and test classifiers for predicting the class of the phenotype of interest.

## Results and discussion

In this section, we first give brief information about the colorectal cancer in human (CRC) and introduce the two stage-specific CRC datasets and the PPI network we use in our experiments. Subsequently, we describe in detail the experimental framework used. After introducing the performance evaluation metrics used, we present our experimental results comparing one-vs-all and all-vs-all discriminative subnetworks, as well as the additive and set-cover based algorithms that are used to discover these subnetworks. Next, we analyze the effect of the network distance parameter (*ℓ*) that adjusts the search space size when growing the subnetworks. Finally, we compare the performance of high-scoring and statistically significant subnetworks in predicting the stages of samples.

### Human colorectal cancer

Colorectal Cancer (CRC) is one of the most common causes of cancer related deaths in the western civilization [12]. Diagnosis of CRC is often difficult as the symptoms appear only at the advanced stages of the disease. Moreover, early diagnosis is very critical as the survival rate changes dramatically with the stage of the cancer. In fact, 5 year survival rates when diagnosis is made at the localized stage (cancer is confined in the primary site) and after cancer has metastasized are around 90% and 12% respectively [13]. These observations suggest that, for effective diagnosis, prognosis and treatment, accurate determination of disease stage is crucial.

There are different classification systems for the progression of colorectal cancer. Dukes' famous staging system classifies patients based on how far the cancer is spread [14]. TNM is another staging method providing a more comprehensive framework including information about the size and localization of the tumor, as well as the involvement of lymph nodes [15]. CRC Datasets we use in our experiments are classified by Dukes' staging system.

### Datasets

We use two CRC microarray datasets obtained from the Gene Expression Omnibus [16] in our experiments. These datasets that contain labeled CRC samples with Dukes' 4-stage classification are the following:

• GSE14333 contains the expression profiles of 54675 genes in 290 samples.

• GSE5206 contains the expression profiles of the same 54675 genes in 98 samples.

The distribution of the samples in each dataset with respect to CRC stage is shown in Table 1.

**Table 1:** Number of samples labeled with each colorectal cancer stage based on Dukes' 4-stage classification in the datasets used in our experiments.

stage	A	B	C	D	total
GSE14333	44	94	91	61	290

GSE5206	12	32	33	21	98

The human protein-protein interaction data used in our experiments is obtained from NCBI Entrez Gene Database [17]. This database integrates interaction data from several other databases available, such as HPRD, Bi-oGrid, and BIND. We remove the nodes with no interactions to obtain a final PPI network that contains 8959 proteins and 33,528 interactions among these proteins.

### Experimental design

COBALT is fully implemented in Matlab. We use this implementation to perform the following classification experiments:

• *Prediction of disease stage in GSE14333*. Subnetworks discovered using GSE14333 are used to predict the stages of samples in the same dataset in a *10-fold cross validation *setting. Samples in each phenotype class are randomly separated into ten similar-sized groups. In each iteration, one of the groups in each class is chosen to be the test data and the rest of the data is used to train the classifier.

• *Prediction of disease stage in GSE5206*. Subnetworks discovered using GSE14333 are used to predict the stages of samples in GSE5206. In this cross-classification setting, the classifier is trained on GSE14333 and tested on the other dataset, GSE5206.

For both of these settings, we use a naive Bayesian classifier provided by Matlab's **classify **function. Using other classifier options provided by Matlab's classifier procedure only marginally effects the results (data not shown).

### Classification performance

In order to obtain a comprehensive picture of the performance of different approaches, we list the precision and recall of the classification experiments for each phenotype class separately. *Precision *refers to the percentage of the correct predictions over all samples predicted belonging to the respective CRC stage, whereas *recall *refers to the percentage of the correctly predicted samples over all samples that are clinically diagnosed to belong the respective CRC stage. Please note that we set the network distance parameter ℓ = 3 in all experiments unless otherwise noted, since it provides the best performance as shown in the next section. When quantizing the expression values of a gene over all samples using Equation 2, we set *α *= 2 as the threshold parameter for the gene's dysregulation in a sample to be considered positively or negatively covering.

We compare COBALT with our implementation of the two additive greedy approaches explained in detail in the Methods section, namely *additive_ova _*and *additive_ava_*. *additive_ova _*and *additive_ava _*refer respectively to the algorithms that aim to identify one-vs-all and all-vs-all subnetworks by greedily maximizing the discriminative power of the subnetworks $\left(\Delta_{\text{one}-\text{vs}-\text{all}}\;\text{and}\;\Delta_{\text{all - vs - all}}\right).$ In the first set of experiments, we use the 10-fold cross validation framework for prediction of disease stage of samples in GSE14333, using the high-scoring subnetworks extracted as features from the same dataset, *i.e*., we use the top scoring positive and negative subnetworks for each stage in COBALT (setting *k *= 1), top 2 subnetworks for each stage in *additive_ova _*and top 8 subnetworks for *additive_ava _*as features (a total of 8 features used in each method).

**Figure 3:**
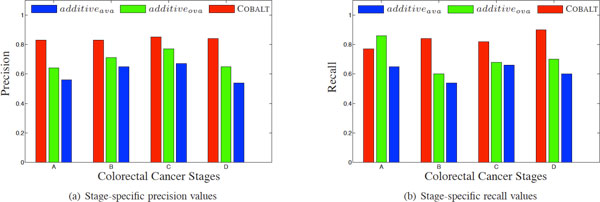
**Precision and recall values for each stage in prediction of stages of samples in GSE14333**. Precision and recall values for each stage in prediction of stages of samples in GSE14333 in a 10-fold cross validation framework are shown in (a) and (b) respectively. COBALT outperforms the additive approaches in terms of precision for all CRC stages. It also provides better recall values for all CRC stages except for Stage A.

As shown in Figure 3, COBALT provides better performance compared to both of the additive approaches in terms of the precision in prediction for all CRC stages. It also provides better recall values for all CRC stages except for samples in Stage A. COBALT achieves 0.84 weighted average precision over all stages where as *additive_ova _*and *additive_ava _*respectively achieve 0.71 and 0.62 precision. Similarly, COBALT outperforms others by achieving 0.84 weighted average recall over all stages where as *additive_ova _*and *additive_ava _*respectively provide 0.69 and 0.60 recall.

#### The effect of the PPI network on classification performance

In this section, we discuss the effect of the PPI network in the classification performance of subnetworks identified by COBALT. Since the use of the PPI network confines the search space to functionally related groups of proteins, these experiments provide insights into whether these functional constraints also improve the biological reproducibility and the utility of identified stage-specific subnetworks. For this purpose, we systematically evaluate the classification performance of the subnetworks for varying network distance parameter (*ℓ*) that adjusts the search space size when growing the subnetworks using COBALT. We also compare the subnetworks identified by the network-guided algorithm with groups of genes that are identified by using the same algorithm in a network-free fashion. The set-cover based algorithm implemented by COBALT is quite efficient, therefore a network-free search for stage-specific groups of proteins is feasible.

**Figure 4:**
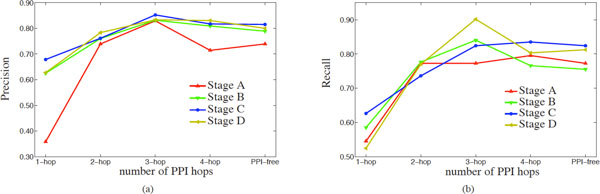
**Precision and recall plots of each stage with respect to different values of the network distance parameter ***ℓ*****. Precision and recall plots of each stage, in prediction of CRC stages of samples in GSE14333 in a 10-fold cross validation framework are shown in (a) and (b) respectively, with respect to different values of the network distance parameter *ℓ*, as well as the PPI-free approach. Setting ℓ = 3 provides the best performance for most of the cancer stages in terms of both precision and recall, also providing a smaller search space compared to ℓ = 4 and the PPI-free approach.

In the PPI network free approach, the next protein to be added to the subnetwork does not need to be in a certain proximity (*i.e*., ℓ is effectively set to ∞) to the proteins already in the subnetwork. This increases the search space for the algorithm, thus making it infeasible for most of the state-of-the-art algorithms to perform some complex analyses such as statistical significance computations. The effect of the parameter ℓ on the classification performance for each CRC stage is shown in terms of precision and recall in Figures 4 (a) and 4(b) respectively. As seen in the figures, the classification performance (hence the reproducibility) of identified subnetworks improves as PPI network neighborhood is defined more flexibly. This is expected, since the PPI network is incomplete, thus consideration of indirect interactions accounts for missing interactions to a certain extent. However, as the search diameter reaches 3, the classification performance saturates and adding more flexibility to the search does not improve performance any more. This observation suggests that incorporation of PPI networks is useful for increased efficiency of the search, as well as identification of more reproducible subnetworks. Thus we set ℓ = 3 as it is the most reasonable choice in terms of both the classification performance and the computational efficiency of the algorithms.

#### The effect of using statistically significant subnetworks on classification performance

In this section, we compare the classification performance of high-scoring subnetworks to that of statistically significant subnetworks. On the GSE14333 dataset, COBALT identifies 9 statistically significant subnetworks with 139 unique genes (please see Additional File 1 for the list of genes and the covered stage of colorectal cancer for these 9 subnetworks). Using these subnetworks as features, we predict the stage of the samples in GSE14333 in a 10-fold cross validation framework as previously explained. We choose the same number of features with top $(\Delta_{\text{one - vs - all}}\left(S\right)$. score and compare the classification performance of both approaches. Stage-specific precision and recall values for both approaches are shown in Table 2. As seen in the table, utilizing statistical significance computations when choosing features improve performance for predicting the stages of patients in GSE14333, with less number of unique genes used.

**Table 2:** Contingency tables for prediction of CRC stages of samples in GSE14333.

		Predicted Classes				
	
	stage	A	B	C	D	recall
**Actual Classes**	A	37	3	0	4	0.84
	
	B	3	74	11	6	0.78
	
	C	5	5	77	4	0.84
	
	D	4	7	3	47	0.82

	**precision**	0.75	0.83	0.84	0.77	

		**Predicted Classes**				
	
	stage	A	B	C	D	**recall**

	A	38	1	2	3	0.86
	
**Actual Classes**	B	4	75	8	7	0.79
	
	C	3	2	83	3	0.91
	
	D	2	3	4	52	0.85

	**precision**	0.80	0.92	0.85	0.80	

Contingency tables for prediction of CRC stages of samples in GSE14333 with COBALT using (a) 9 statistically significant features composed of 139 unique genes, (b) 9 features with top mutual information scores composed of 144 unique genes. The weighted average precision scores are 0.86 and 0.81 for (a) and (b) respectively. Similarly, weighted average recall values are 0.85 and 0.81 respectively for (a) and (b).

In the cross-classification framework, we use the statistically significant features identified in GSE14333 to predict the classes of samples in the GSE5206 dataset, *i.e*., the classifier is trained using GSE14333 and tested on GSE5206. The stage-specific precision and recall values are shown in Table 3. The weighted average precision and recall values are 0.57 and 0.56 respectively.

**Table 3:** Contingency table for prediction of CRC stages of samples in GSE5206 using the statistically significant features identified from GSE14333.

		Predicted Classes				
	
	stage	A	B	C	D	recall
	A	9	2	1	0	0.75
	
**Actual Classes**	B	3	18	8	3	0.56
	
	C	5	3	20	5	0.60
	
	D	4	3	6	8	0.38

	**precision**	0.42	0.69	0.57	0.50	

## Conclusions

In this article, we have proposed two alternate formulations of the discriminative power of subnetworks when working on multi-class phenotypes, namely, one-vs-all and all-vs-all. We then introduced our cover-based algorithm for network-guided disease marker discovery, for identifying subnetworks with one-vs-all discriminative power. Moreover, we have introduced a framework for assessing the statistical significance of the identified subnetworks. Systematic experiments on real multi-staged CRC datasets show that the proposed algorithm outperforms the additive algorithms in terms of providing higher precision and recall in prediction of sample stages. The efficient implementation of the cover-based algorithm enabled us to show that using statistically significant subnetworks as features improves classification performance compared to using same number of high-scoring subnetworks (in terms of mutual information with respect to the phenotype vector). We have also shown that guiding the subnetwork discovery search with the PPI network identifies subnetworks that are more informative (in terms of classification power) than the net-works identified without the PPI network. We have also investigated the impact of different values of the network distance parameter, *ℓ*, and concluded that using ℓ = 3 is the most reasonable choice in terms of both classification performance and computational efficiency.

## Competing interests

No competing interests exist.

## Authors' contributions

All authors conceived and formulated the problem; SE and MK conceived and formulated the proposed approach and algorithms; SE implemented and tested the algorithms; RKE provided the expression data; SE and MK drafted the manuscript; all authors reviewed, edited, and approved the final manuscript.

## Supplementary Material

Additional file 1 **List of **9 **statistically significant subnetworks identified for GSE14333 dataset**. All the gene products in the 9 statistically significant subnetworks identified for GSE14333 dataset are listed, as well as the covered colorectal cancer stage and cover direction of the corresponding subnetworks. Please note that these gene products might not be direct neighbours in the PPI network, as we set the network distance parameter ℓ = 3 in the experiments. Click here for file
