# Supplementary material for: Identifying stage-specific protein subnetworks for colorectal cancer
Source: BMC Proc. 2012 Nov 13;6(Suppl 7):S1. doi: 10.1186/1753-6561-6-S7-S1 (PMC3504924; doi:10.1186/1753-6561-6-S7-S1)
Supplement: Additional file 1 — List of 9 statistically significant subnetworks identified for GSE14333 dataset. All the gene products in the 9 statistically significant subnetworks identified for GSE14333 dataset are listed, as well as the covered colorectal cancer stage and cover direction of the corresponding subnetworks. Please note that these gene products might not be direct neighbours in the PPI network, as we set the network distance parameter ℓ = 3 in the experiments. [file 1753-6561-6-S7-S1-S1.PDF]

Additional File 1

Subnetwork 1	Subnetwork 2	Subnetwork 3	Subnetwork 4	Subnetwork 5	Subnetwork 6	Subnetwork 7	Subnetwork 8	Subnetwork 9
SMEK2	LMO3	XPC	ZNF398	SIGIRR	EPHB4	SEC61A2	ONECUT1	AGAP2
ADAM17	SERPINI1	CD27	TRIM6	DIMT1L	CCNB3	AJAP1	GPR124	PEX10
WSB2	MST1R	NUP37	PPP1R9B	PTCD3	SAPS1	VEZT	U2AF2	CLSTN3
STRN4	COPS8	CD163	XBP1	AQP3	SYTL4	NDE1	SIRPA	NCSTN
C6orf15	ZBED1	PECAM1	ARHGEF9	KIT	LRP1B	DUSP22	COL4A3	MED23
UBR5	HIST1H2BN	RALGPS2	RTN3	IL4	SNX17	TAOK3	NTRK3	DDB1
SNAPC3	BCAN	ARL2	SENP2	GTF2H5	NUDT14	TFG	ZNF581	COMMD1
PRRX1	ZNF335	NEO1	SARS	GANAB	DYRK1A	NPLOC4	C8orf48	KIF1A
HOXD10	PARP4	MOBKL3	DUSP22	MAP2	PRKCA	NRG2	C16orf48	BBS2
PLOD3	YWHAQ	OAZ3	SHC3	SP140	IFITM1	FMNL1	MTOR	GRM4
SERPINB6	CRCT1	STAT3	CBX3	COL4A3BP	SH3RF1	ELK4		LRFN1
GRM3	GYS1		STAM	PDIA3	PTEN	ZRANB1		LRSAM1
MSX1	NOS1AP		DLX2	TTBK1	ZAP70	CD38		G3BP1
CABP1	SFRS4		ABI1	AXIN2	HRAS	SNTB1		SMC1A
AQP5	DNTTIP1			HEATR2	NAT1	VPS37C		HIPK1
CD177	NXF3			MEP1A		HIST1H2AG		EPHB2
TNNT1	EXD3			PRIMA1		LRP1		
DOK5	IRF1					CTRL		
A2M	ALX1							
stage C positive	stage B positive	stage A positive	stage D positive	stage C positive	stage D negative	stage B negative	stage A negative	stage C negative
